# EU-HTA Guidance for Clinical Validity: Misconceptions and Flawed Processes

**DOI:** 10.3390/jmahp13030033

**Published:** 2025-07-15

**Authors:** Mondher Toumi, Bruno Falissard, Asma Jouini, Samuel Aballéa, Laurent Boyer, Pascal Auquier

**Affiliations:** 1 CEReSS/UR3279—Health Services Research and Quality of Life Center, Aix-Marseille University, 13385 Marseille, France; laurent.boyer@univ-amu.fr (L.B.); pascal.auquier@univ-amu.fr (P.A.); 2 InovIntell, 215 rue du Faubourg St Honoré, 75008 Paris, France; 3 CESP, INSERM U1018, Université Paris-Saclay, 94800 Villejuif, France; bruno.falissard@gmail.com; 4 Clever Access, Tunis 1053, Tunisia; asma.jouini@clever-access.com; 5 InovIntell Co., Ltd., 3023 GJ Rotterdam, The Netherlands; sab@inovintell.com

This review of the scope of the European Health Technology Assessment (EU HTA)’s guidance on clinical trial validity in its randomized controlled trials (RCTs) highlights several key issues that undermine its practical application and effectiveness, including misconceptions, errors, and inconsistencies [1]. These flaws are attributable to the absence of a systematic literature review (SLR), a lack of transparency, a lack of accountability, and a lack of consideration of evidence-based medicine (EBM) principles, as mandated by the EU HTA Regulation (EU HTAR) [2,3].

A key concern is the contradiction between the EU HTAR’s prohibition of judgment [3] and the inherent need for judgment in clinical trial evaluations [4,5]. Assessors must determine comparative effectiveness [3], which requires evaluating study design, inclusion criteria, outcomes, and their impact on validity and statistical precision [6]. Prohibiting judgment leads to flawed assessments [6,7], as bias identification—a core aspect of this guidance—relies on it [4,8,9]. Banning judgment undermines clinical evidence assessments.

A significant issue with this guidance is its misinterpretation and inaccurate representation of fundamental concepts within clinical research, including internal and external validity. The guidance defines internal validity as a study’s freedom from bias [1]. However, internal validity is not just about bias control; it is about establishing causality between an intervention and observed outcome [10,11,12]. Even in randomized trials, confounder imbalances and population heterogeneity can weaken causal inferences [10,11,12].

Moreover, the guidance erroneously suggests that biases exclusively affect the internal validity of assessments, but in reality, multiple biases can have significant implications for the external validity of assessments [1]. Additionally, the guidance fails to acknowledge the trade-offs between internal and external validity. Highly controlled trials (high internal validity) may lack real-world applicability (low external validity), while pragmatic trials prioritize generalizability at the cost of causal certainty [12,13,14]. Tools like PRECIS-2 help to balance these dimensions [15,16], yet the guidance offers no such framework. Expecting a single trial to maximize both types of validity alongside statistical precision contradicts methodological reality.

The guidance suggests that external validity can be sufficiently evaluated using qualitative methods on a case-by-case basis [1]. However, this approach may not fully acknowledge the complexity of external validity and the multiple factors—beyond PICO (Population, Intervention, Comparator, Outcome)—that influence the generalizability of study results [17]. Several biases specifically affect external validity [9,17,18]. Using standardized tools or checklists to guide the assessment of external validity, would ensure a more rigorous and transparent evaluation [9].

Although the guidance states that PICO differences affect external validity, internal validity is also PICO-dependent but is still assessed centrally [19]. A more practical approach would be to assess external validity at the PICO level while leaving broader contextual considerations to Member States (MSs)’ discretion.

The guidance ignores Type I and II errors and avoids concluding superiority, non-inferiority, or equivalence. Instead, it focuses on whether the tested hypothesis is or is not rejected, which inherently acknowledges these errors and conclusions [1]. This exclusion appears unreasonable as this factual information is important to HTAs [1,20]. The arguments for prohibited judgment cannot justify this recommendation.

Minimum Clinical Important Difference (MCID) is not addressed while it should be consistent across all MS and managed within the Joint Clinical Assessment (JCA) [1]. It may be reasonable to ask Health Technology Developers (HTDs) to document MCID for critical endpoints.

The guidance advocates for intention-to-treat (ITT) analysis to control for attrition, but it fails to consider that ITT may not be suitable for progressive diseases [21].

Several recommendations within the guidance, such as the selection of Risk of Bias (RoB) 1 over the updated version RoB 2 for bias assessment [1,8] and the assessment method recommended for external validity, are arbitrary [1]. Similarly, the exclusion of Bayesian adaptive trials and pragmatic trials from the guidance limits the relevance of this guidance while these trial designs can provide valuable perspective for HTA decision [1].

Conclusions:

The EU HTA guidance on clinical trial validity is fraught with limitations, stemming from a lack of SLR-based recommendations, a lack of transparency, a lack of accountability, failure to adhere to EBM principles, and the prohibition of judgment [2,3], leading to significant misconceptions and errors. A thorough revision of the guidance is needed, incorporating SLRs to justify methodological choices, expert consultations, greater transparency, and compliance with EBM as requested in the EU HTAR.

Evaluating clinical trials is complex and involves various cognitive skills and judgments [22]. Policymakers likely prohibit judgment in the EU HTAR for good reason. This likely reflects the belief that appraising the value of an intervention and its positioning should be managed by MSs to address national specificities. It is unlikely that this rule is intended to restrict assessors from exercising their scientific judgment. This requires clarification. Prior to assessing comparative effectiveness certainty, the HTA coordination group and the European Commission DG Santé should resolve any methodological uncertainties from their guidance.

## Abbreviations

The following abbreviations are used in this manuscript:

EBM	Evidence-Based Medicine
EU	European Union
EU HTA	European Health Technology Assessment
EU HTAR	European Health Technology Assessment Regulation
HTA	Health Technology Assessment
HTDs	Health Technology Developers
ITT	Intention To Treat
JCA	Joint Clinical Assessment
MCID	Minimum Clinically Important Differences
MS	Member States
PICO	Population, Intervention, Comparator, Outcome
PRECIS	PRagmatic Explanatory Continuum Indicator Summary
RoB	Risk of Bias
SLR	Systematic Literature Reviews
